# Forecasting Carbon Dioxide Price Using a Time-Varying High-Order Moment Hybrid Model of NAGARCHSK and Gated Recurrent Unit Network

**DOI:** 10.3390/ijerph19020899

**Published:** 2022-01-14

**Authors:** Po Yun, Chen Zhang, Yaqi Wu, Yu Yang

**Affiliations:** 1 School of Economics and Management, Hefei University, Hefei 230601, China; 2 School of Management, Hefei University of Technology, Hefei 230601, China; sm.zhangchen@hfut.edu.cn; 3 School of Economics, North Minzu University, Yinchuan 750021, China; yq111099@mail.hfut.edu.cn; 4 School of Economics and Management, Anhui Jianzhu University, Hefei 230601, China; yangyu@ahjzu.edu.cn

**Keywords:** carbon price forecasting, time-varying, high-order moment, NAGARCHSK, gate recurrent unit network

## Abstract

The carbon market is recognized as the most effective means for reducing global carbon dioxide emissions. Effective carbon price forecasting can help the carbon market to solve environmental problems at a lower economic cost. However, the existing studies focus on the carbon premium explanation from the perspective of return and volatility spillover under the framework of the mean-variance low-order moment. Specifically, the time-varying, high-order moment shock of market asymmetry and extreme policies on carbon price have been ignored. The innovation of this paper is constructing a new hybrid model, NAGARCHSK-GRU, that is consistent with the special characteristics of the carbon market. In the proposed model, the NAGARCHSK model is designed to extract the time-varying, high-order moment parameter characteristics of carbon price, and the multilayer GRU model is used to train the obtained time-varying parameter and improve the forecasting accuracy. The results conclude that the NAGARCHSK-GRU model has better accuracy and robustness for forecasting carbon price. Moreover, the long-term forecasting performance has been proved. This conclusion proves the rationality of incorporating the time-varying impact of asymmetric information and extreme factors into the forecasting model, and contributes to a powerful reference for investors to formulate investment strategies and assist a reduction in carbon emissions.

## 1. Introduction

Dramatic increase in greenhouse gas emissions directly leads to the aggravation of negative environmental externality. The emission of pollutants such as carbon dioxide is a serious threat to human health, and it is unacceptable that the pollution of this harmful gas will continue in the foreseeable future. According to the report of the Word Bank, the global carbon emissions in 2018 were 2.1 times the 1960 level, the per capita emissions increased by nearly 67%, and the per capita energy consumption increased by nearly 60% (data from author’s calculation based on the Wind database). Economic growth, CO_2_ emissions and energy consumption are complementary [1,2]. Based on the scientific report released by the National Oceanic and Atmospheric Administration (NOAA) of the United States in 2020, the global CO_2_ concentration was 280 ppm at the beginning of the first industrial revolution, that is, the CO_2_ quality accounted for 2.8% of the global atmospheric quality. In 2017, this data increased to 400 ppm, and in 2019, it increased to 415 ppm. The continuous growth of carbon dioxide emissions not only leads to the rise of global temperature, triggers sea-level rise, aggravates glacier melting and other severe environmental problems, but also threatens human health, and ultimately affects the sustainability of global economy and human civilization. Therefore, it has become an urgent task for human society to effectively curb the global climate problems and reduce greenhouse gas emissions.

The establishment of carbon market is a market-oriented means for the international community to solve climate problems and reduce pollution emissions. Based on the “Kyoto Protocol” signed in 2005 and the “Paris Agreement” passed in 2015, the carbon allowance assets traded in the carbon market have commodity and financial attributes, and there exists three exclusive characteristics that cannot to be ignored compared with other capital markets. The first is the asymmetric distribution of market returns, the tail distribution has the characteristics of left deviation, and the skewness is negative [3,4,5]. The second is the high sensitivity to policy events or external events [6]. For example, the policy implementation of banning interterm storage of carbon quotas led to a serious decline in European carbon price at the end of 2007; the fall in carbon price caused by the expiration of the second phase of emission reduction in Europe at the end of 2012; the outbreak of COVID-19 virus led to global economic downturn and triggered a sharp drop in carbon price. The third is the time-varying characteristics of carbon price volatility [7,8,9]. Therefore, the research into carbon price prediction and pricing models need to reflect the above three indispensable characteristics. The price mechanism is the core of the carbon market to promote emission reduction of the whole society. Consequently, studying the pricing mechanism of the carbon market in this paper can better serve the emission reduction practice of entity enterprises and create a healthier social environment.

The structure of this paper is as follows: the second part is the literature review; the third section analyzes the econometric model; the fourth section is the empirical analysis and discussion; the last part summarizes the conclusion and the prospects.

## 2. Literature Review

Existing research methods on carbon price forecasting mainly focus on two aspects: one is the volatility modeling technology and the other is artificial intelligence-integrated technology.

### 2.1. Volatility Modeling Technology

As for volatility modeling technology, Byun and Cho [10] pointed out that the GARCH family model could better fit the carbon future returns than other volatility models. Conducting the asymmetric threshold GARCH model, Chevallier [11] concluded that the stock and bond market variables could effectively explain the asymmetric volatility of carbon future returns. Based on the autoregressive, comprehensive, moving average model, Dhamija et al. [12] found that the asymmetric ARIMA-GARCH model can fit the conditional return and variance of European carbon price. Using the multi-GARCH model, Oberndorfer [13] stated that the EUA (European Union Allowance, EUA) price was positively correlated with the electricity stock return, and the stock market return did not cause EUA market volatility. Based on the ARCH regression model, the crude oil, natural gas and coal returns have a significant effect on carbon price [14,15]. Testing the EGARCH model, Chevallier [16] maintained that the abnormal events, policy factors, compliance events and uncertainty after the Kyoto Protocol are evidence of the instability of carbon price. The time-varying GARCH model with generalized nonlinear parameters can effectively fit the carbon price for prediction [16]. Designing bilaterally modified variables, Ren et al. [17] point out that the AR-GARCH model can reveal the impacts of regulatory update events on the Chinese carbon market. Employing the dynamic nonlinear (DMA) model, Koop et al. [18] found that the pricing precision of the DMA model is superior to the time-varying parameter regression model (TVP). The European carbon price is characterized by heterogeneous volatility, the prediction performance of the GARCH model based on Markov regime switching is better than other GARCH models [19].

### 2.2. Artificial Intelligence-Integrated Technology

The volatility modeling technology represented by the GARCH family model usually requires the carbon price being subject to strict parameter assumptions and tail distribution, which means the application of the model has great limitations [18]. Artificial intelligence-integrated technology with the advantages of mapping nonlinear relations and without considering the tail distribution has been widely used in carbon price forecasting research. The BP neural network model with high-frequency data has a more accurate prediction performance on the CER (Certified Emission Reduction, CER) price than the GARCH family model [20]. Tiwari et al. [21] found that the time-varying Markov switching copula model can provide evidence of a time-varying tail-dependence structure, and AI (artificial intelligence) is an effective means to capture carbon price. The finite distributed lag (FDL) model based on a genetic algorithm (GA) has better performance on predicting carbon price than other GARCH models [22]. Based on the idea of ensemble learning, the EMD model (Empirical Mode Decomposition, EMD) is used to extract the intrinsic mode function (IMF) that represents the different coexisting oscillation modes of carbon series [23,24,25], and then a hybrid carbon price forecasting model integrating the variational mode decomposition (VMD) and optimal combination forecasting model (CFM) is constructed, the results suggesting the superiority of the proposed hybrid model for carbon price forecasting [26,27]. Conducting the EMD method, Wang et al. [28] proposed a new random forest-based nonlinear ensemble paradigm for carbon price prediction and proved the model’s superiority in European carbon price forecasting. Furthermore, the hybrid carbon price forecasting model, for example the multiobjective grasshopper optimization algorithm model proposed by Hao et al. [29] and the wavelet least-square support vector machine (WLSSVM) model carried by Sun et al. [30] have been proven to have strong superiority and accuracy in carbon price prediction. Different from the prediction of EMD-type models, the LSTM (Long and Short-Term Memory network) model does not need to decompose the data frequency, and shows advantages in capturing the long-term lag return characteristics based on the special gate structure of forget gate, input gate and output gate [31,32]. The application of the LSTM model in predicting stock market index has stronger accuracy and robustness than other exponential smooth models and the ARIMA model [33,34]. Employing the models of ARIMA, CNN, GARCH and LSTM to extract the linear characteristics, hierarchical data structure, long memory characteristics and volatility characteristics of carbon return, respectively, the conclusion suggests that the hybrid model of ARIMA–CNN–LSTM and GARCH-LSTM contribute a lower prediction error [35,36]. Based on similar modeling ideas, the integrated models of EMD–LSTM and that composed of total average EMD with LSTM (MEEMD–LSTM) have also proven to have significant superiority in carbon price prediction [37,38].

The above literature provides valuable references for this paper. However, the most obvious defect is that the existing literature ignores the time-varying impact of market asymmetric information and extreme shock factors on carbon premium from the perspective of high-order moment (skewness and kurtosis). More importantly, the time-varying high-order moment characteristics have been ignored. In fact, studies have proven that the financial assets of low-order moment information cannot fully reflect the actual financial return distribution [39]. The innovation and contribution of this study is to construct a new hybrid carbon pricing model, NAGARCHSK-GRU, that reveals the time-varying high-moment volatility characteristics of carbon price. The proposed NAGARCHSK-GRU price-forecasting model combines the advantages of the NAGARCHSK model in parameter estimation of the time-varying, high-order moment characteristics and the superiority of GRU (Gated Recurrent Unit, GRU) network in nonlinear fitting and forecasting. The purpose for integrating the models of NAGARCHSK and GRU network is to improve the robustness and generalization ability of the proposed pricing model, and then to provide certain technical support for market participants to capture price information and predict carbon price.

## 3. Econometric Modeling

Based on the classical GARCH models, this paper first constructs constant and time-varying, high-order moment carbon price volatility methods to estimate the parameters of the proposed pricing model. Secondly, the multilayer GRU network model is designed to realize the nonlinear prediction based on the time-varying, high-order moment parameters estimated by the NAGARCHSK model.

### 3.1. High-Order Moment Volatility Model

#### 3.1.1. Constant High-Order Moment Model

The constant high-order moment model assumes that the third-order moment skewness and the fourth-order moment kurtosis have no impact on the first-order moment return of carbon price, but assumes that they are constant. The common constant high-moment model is the GARCHSK (*q*1,*p*1;0,0;0,0) model with the constant high-order moment term. During modeling of the carbon price, we use the AR (R) model to describe the autocorrelation process of carbon price series, assuming the return series follows a first-order lag AR ® process:

(1) $$R_{t}=\rho R_{t-1}+h_{t}^{1/2}\xi_{t}$$

where, $h_{t}^{1/2}$ is the conditional variance of carbon return; $\xi_{t}$ means the conditional return item; ρ indicates the autocorrelation coefficient, and $\xi_{t}∼N(0,1)$.

For modeling the conditional variance *h_t_* process that with the characteristics of volatility clustering and asymmetric distribution, this paper uses the GARCH model and its derivative models to estimate the parameters of the proposed carbon pricing model. As the first-order GARCH model can simulate the financial return volatility, we introduce the following common forms of conditional variance based on the GARCH (1,1) model.

Conditional variance of GARCH (1,1) is:

(2) $$h_{t}=\beta_{0}+\beta_{1}\varepsilon_{t-1}^{2}+\beta_{2}h_{t-1}$$

Conditional variance of TGARCH (1,1) is:

(3) $$h_{t}^{1/2}=\beta_{0}+\beta_{1}\left|\varepsilon_{t-1}\right|+\beta_{2}h_{t-1}+\beta_{3}\upsilon_{t-1}\left|\varepsilon_{t-1}\right|$$

Conditional variance of NAGARCH (1,1) is:

(4) $$h_{t}=\beta_{0}+\beta_{1}{\left(\varepsilon_{t-1}+\beta_{3}h_{t-1}^{1/2}\right)}^{2}+\beta_{2}h_{t-1}$$

where, $\beta_{0}$ represents the constant term of the variance equation, skewness equation and kurtosis equation; $\beta_{1}$ and $\beta_{2}$ denote the ARCH and GARCH term coefficients of the high-order moment equation, respectively; ε represents the residual term; $\beta_{3}$ means the leverage coefficient, reflecting the impact of asymmetric information on the carbon returns; $\upsilon_{t-1}$ is a dummy variable that controls the impact direction of asymmetric information, when $\varepsilon_{t-1}$ < 0, $\upsilon_{t-1}$ = 1;$\varepsilon_{t-1}$ > 0, $\upsilon_{t-1}$ = 0.

#### 3.1.2. Time-Varying High-Order Moment Model

The constant high-order moment model regards the third-order moment skewness and fourth-order moment kurtosis as fixed constants and ignores the financial asset distribution characterization of leptokurtosis and fat-tail caused by the market asymmetric information and extreme factors, which make it difficult to meet the real asset volatility-modeling requirements. Therefore, this paper considers the third-order skewness and fourth-order moment kurtosis attributes with the exclusive features of time-varying volatility, so as to describe the shock of market asymmetric information and policy factors on carbon price. The specific form of the GARCHSK (*q*1,*p*1;*q*2,*p*2;*q*3,*p*3) model, considering the volatility of time-varying conditional variance, conditional skewness and conditional kurtosis, is as follows:

(5) $$\left\{ \begin{array}{l} R_{t}=\rho E_{t-1}(R_{t})+\varepsilon_{t}=\mu_{t}+h_{t}^{1/2}\xi_{t};\xi_{t}|I_{t-1}∼F_{n}(0,1,s_{t},k_{t})\\ h_{t}=\beta_{0}+\sum_{i=1}^{q_{1}}\beta_{1,i}\varepsilon_{t-i}^{2}+\sum_{j=1}^{p_{1}}\beta_{2,j}h_{t-j}\\ s_{t}=\gamma_{0}+\sum_{i=1}^{q_{2}}\gamma_{1,i}\xi_{t-i}^{3}+\sum_{j=1}^{p_{2}}\gamma_{2,j}s_{t-j}\\ k_{t}=\delta_{0}++\sum_{i=1}^{q_{3}}\delta_{1,i}\xi_{t-i}^{4}+\sum_{j=1}^{p_{3}}\delta_{2,j}k_{t-j} \end{array}\right.$$

The specific form of the NAGARCHSK (*q*1,*p*1;*q*2,*p*2;*q*3,*p*3) model with leverage effect that considers the volatility of the time-varying, high-order moment, is as follows:

(6) $$\left\{ \begin{array}{l} R_{t}=\rho E_{t-1}(R_{t})+\varepsilon_{t}=\mu_{t}+h_{t}^{1/2}\xi_{t};\xi_{t}|I_{t-1}∼F_{n}(0,1,s_{t},k_{t})\\ h_{t}=\beta_{0}+\sum_{i=1}^{q_{1}}\beta_{1,i}{\left(\varepsilon_{t-1}+\beta_{3,i}h_{t-i}^{1/2}\right)}^{2}+\sum_{j=1}^{p_{1}}\beta_{2,j}h_{t-j}\\ s_{t}=\gamma_{0}+\sum_{i=1}^{q_{2}}\gamma_{1,i}\xi_{t-i}^{3}+\sum_{j=1}^{p_{2}}\gamma_{2,j}s_{t-j}\\ k_{t}=\delta_{0}+\sum_{i=1}^{q_{3}}\delta_{1,i}\xi_{t-i}^{4}+\sum_{j=1}^{p_{3}}\delta_{2,j}k_{t-j} \end{array}\right.$$

where, $I_{t-1}$ represents the information set when the carbon return volatility reaches the time of *t* − 1; $E_{t-1}(R_{t})$ is the corresponding conditional expected return that can be obtained steadily without risk impact under the certain $I_{t-1}$ information set. The form AR(1) is used to depict the autoregressive carbon return process. $F_{t}(0,1,s_{t},k_{t})$ represents the fourth-order moment distribution type of the carbon return series based on the classical GARCH(1,1) model, and we can obtain $E_{t-1}(\xi_{t})=0$, $E_{t-1}(\xi_{t}^{2})=1$, $E_{t-1}(\xi_{t}^{3})=s_{t}$, $E_{t-1}(\xi_{t}^{4})=k_{t}$; $s_{t}$ and $k_{t}$ represents the skewness and kurtosis corresponding to standardized residual $\xi_{t}=h_{t}^{-1/2}\varepsilon_{t}$. $\beta_{0},\beta_{1},\beta_{2},\beta_{3}$ denotes the coefficient of the conditional variance equation; $\gamma_{0},\gamma_{1},\gamma_{2}$ represents the coefficient of the conditional skewness equation; $\delta_{0},\delta_{1},\delta_{2}$ means the coefficient of the conditional kurtosis equation. (*q*1,*p*1);(*q*2,*p*2);(*q*3,*p*3) represents the lag order of the conditional variance, conditional skewness and conditional kurtosis equations for capturing the relationship between carbon return and its time-varying, conditional, high-order moment term.

For estimating the parameters of the time-varying, high-order moment model (NAGARCHSK), the Gram–Charlier expansion of normal density function is used and truncates it in the fourth moment. Then, the conditional probability density of the standard error can be obtained under the information set *I*_*t*−1_:

(7) $$\begin{array}{c} f(\xi_{t}|I_{t-1})=g(\xi_{t})\lambda (\xi_{t})/\Gamma_{t}\\ \frac{1}{\sqrt{2\pi }}e^{-\xi_{t}^{2}/2}(1+\frac{s_{t}^{*}}{3!}(\xi_{t}^{3}-3\xi_{t})+\frac{k_{t}^{*}-3}{4!}(\xi_{t}^{4}-6\xi_{t}^{2}+3))/(1+\frac{s_{t}^{*}}{3!}+\frac{k_{t}^{*}-3}{4!}) \end{array}$$

where $\Gamma_{t}=1+\frac{s_{t}^{*}}{3!}+\frac{k_{t}^{*}-3}{4!}$.

Furthermore, the conditional distribution of $\varepsilon_{t}$ is expressed as $h_{t}^{-1/2}f(\xi_{t}|I_{t-1})$, and the log likelihood function is expressed as:

(8) $$LF(\varepsilon_{t}|I_{t-1,}\theta )=-\frac{1}{2}\ln(2\pi )-\frac{1}{2}\ln h_{t}-\frac{1}{2}\xi_{t}^{2}+\ln(\lambda^{2}(\xi_{t}))-\ln(\Gamma_{t})$$

By maximizing the likelihood function above, the consistency estimation of the parameter vector can be obtained, and the parameter estimation results of the conditional mean equation, conditional variance, conditional skewness and conditional kurtosis equations can also be obtained simultaneously. Where $\theta =[\beta ,\gamma ,\delta {]}^{\prime }=[\beta_{0},\beta_{1},\beta_{2},\beta_{3};\gamma_{0},\gamma_{1},\gamma_{2};\delta_{0},\delta_{1},\delta_{2}{]}^{\prime }$ is the parameter vector, representing the parameter to be estimated in the time-varying, high-order moment, carbon price volatility model.

### 3.2. GRU Model

**Figure 1:**
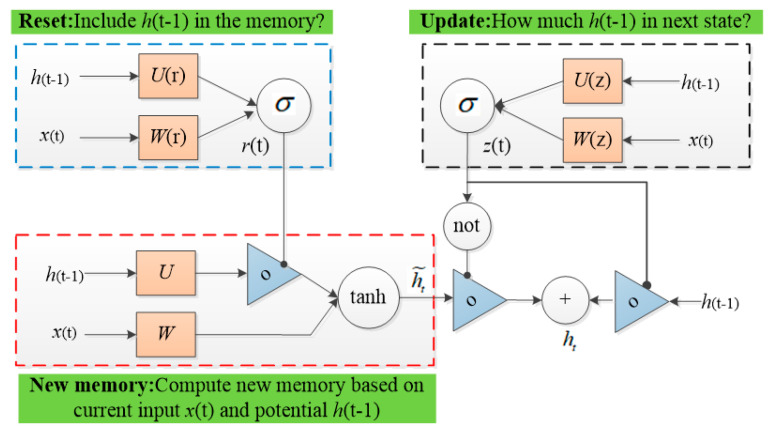
The model training structure of the GRU network.

For mapping the nonlinear, time-varying, high-order moment shock of market asymmetric information and extreme events on carbon price, this paper constructs a multilayer GRU (Gated Recurrent Unit, GRU) model to predict and fit the carbon price with the characteristic of the time-varying, high-order moment. Different from the special input gate, forget gate and output gate structure of the LSTM (Long and Short-Term Memory network, LSTM), another feedforward network structure similar to the GRU network, the GRU model is constructed based on the gate structure of the LSTM and composed of update gate and reset gate [40]. The GRU training structure can be showed in Figure 1.

Specifically, the update gate of GRU is combined of the input gate and forget gate of the LSTM network, and this function is used to determine the information to be discarded and the new information to be added. The reset gate determines the forgotten information in the past time series, which can help to capture the short-term dependency of the finance series. Unlike the LSTM model, which relies on the cell units to obtain the long-term information, the GRU network gets rid of the cell state instead of the hidden state, in order to transmit the previous information and obtain the long-term dependency. Although the debate about the model superiority of GRU and LSTM network continues, it is generally accepted that as an effective variant of LSTM network, the structure of the GRU network is simpler and requires fewer parameters and training samples. Therefore, some studies suggest that GRU is more effective than the LSTM model in solving the long dependency problem of RNN networks [41]. According to the above GRU model diagram, the forward propagation process of the GRU network is as follows:

Firstly, the state *h*_*t*−1_ transmitted from the previous network is combined with the input *x*_*t*_ of the current node to obtain the gate structure of the GRU network, that is, the reset gate r and update gate *z*. Where σ means the activation function, which converts the input data to a value in the range of 0–1 to act as a gating signal.

(9) $$r_{t}=\sigma (W_{r}x_{t}+U_{r}h_{t-1})$$

(10) $$z_{t}=\sigma (W_{z}x_{t}+U_{z}h_{t-1})$$

Secondly, after obtaining the gating signal, the reset gate is used to obtain the data after “reset”. If the element value *r*_*t*_ in the reset gate is close to 0, it means the hidden state element related to the reset gate should be set to 0, that is, the hidden state information of the last time should be discarded. Further, the result of element multiplication is linked to the input of current time step, and the candidate hidden state $\tilde{h_{t}}$ is calculated by the activation function tanh, and the element value ranges from −1 to 1. The calculation for candidate hidden state is:

(11) $$\tilde{h_{t}}=tanh(Wx_{t}+r_{t}Uh_{t-1})$$

Finally, the most critical process of training the GRU model is the update memory stage. The update gate *z_t_* controls the forgotten information of the hidden layer *h*_*t*−1_ at the previous moment, and the new hidden layer information $\tilde{h_{t}}$ needs to be added at the current moment. The update gate *z_t_* is expressed as:

(12) $$h_{t}=(1-z_{t})h_{t-1}+z_{t}\tilde{h_{t}}$$

It is worth noting that the value of updated gating *z_t_* is in a range from 0 to 1. The closer the gating value is to 1, the more data there is to be remembered, while the closer it is to 0, the more information is forgotten. The GRU model can realize data forgetting and memory at the same time by using update gating *z_t_*, unlike the LSTM model that requires multiple gating.

### 3.3. NAGARCHSK-GRU Model 

The proposed hybrid carbon price forecasting model combines the advantages of NAGARCHSK and GRU neural networks. Firstly, the NAGARCHSK model is better than the constant high-order moment models and other time-varying, high-order moment models in fitting the carbon price series with time-varying, high-order moment volatility characteristics. Therefore, we select the NAGARCHSK model to estimate the time-varying parameters of carbon price. 

**Figure 2:**
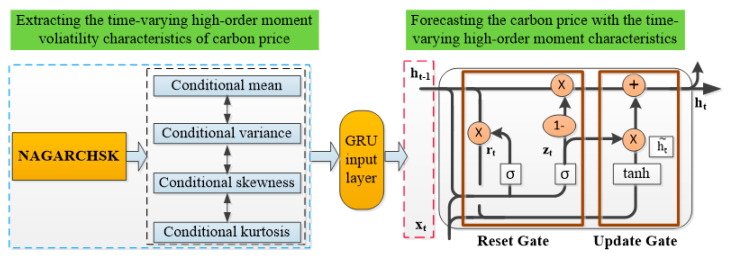
The structuring idea of the carbon price-forecasting hybrid model of NAGARCHSK–GRU.

Secondly, we use these estimated parameters as network inputs, and use the GRU neural network to train the time-varying, high-order moment volatility characteristics of carbon price for improving the prediction accuracy. The basic idea of constructing the proposed NAGARCHSK-GRU carbon-forecasting model shown in Figure 2.

### 3.4. Evaluation Criteria and the Benchmark Model

For evaluating the prediction performance of the proposed time-varying, high-order moment carbon-pricing model, this paper adopts the following criteria to measure the model performance.

(13) $$RMSE=\sqrt{\frac{\sum_{i=1}^{T}{\left(y_{i}-{\hat{y}}_{i}\right)}^{2}}{T}}$$

(14) $$MAE=\frac{1}{T}\sum_{i=1}^{T}\left|y_{i}-{\hat{y}}_{i}\right|$$

(15) $$MAPE=\frac{1}{T}\sum_{i=1}^{T}\left|\frac{y_{i}-{\hat{y}}_{i}}{y_{i}}\right|$$

(16) $$DA=\frac{1}{T}\sum_{i=1}^{T-1}a_{i}\;\mathrm{where}\;a_{i}=\left\{ \begin{array}{l} 1,if(y_{i+1}-y_{i})\times ({\hat{y}}_{i+1}-y_{i})>0\\ 0,otherwise \end{array}\right.$$

where $Y=\left\{y_{1},y_{2},\cdots ,y_{T}\right\}$ represents the carbon return series; $\hat{Y}=\left\{{\hat{y}}_{1},{\hat{y}}_{2},\cdots ,{\hat{y}}_{T}\right\}$ represents the prediction return. *T* is a time series variable.

The values of root-mean-square error (RMSE), mean absolute error (MAE) and mean absolute percentage error (MAPE) range from 0 to 1, and a larger value means the deviation between predicted return and real return is greater, and the model performance is worse. The correct investment direction prediction can help investors make more valuable decisions. This paper uses DA (direction accuracy) index to measure the consistency probability of market trend and investors’ prediction direction. The larger DA value means the predicted value of carbon return is closer to investors’ psychological expectation.

For assessing the performance of the proposed pricing model, this paper also selects the following aggressors as the comparison benchmarks. The first one is the BP (back propagation network) model with the advantage of nonlinear mapping. The second is GBR (gradient boosting regression) model, which is a kind of integrated learning method. The third is MLP (multilayer perceptron); the parameter optimization of the MLP can improve the nonlinear mapping and carbon price-prediction accuracy. The fourth is the RNN (recurrent neural network) model, which is an artificial neural network with a tree structure, that has significant advantages in forecasting carbon price. The fifth is the LSTM (long and short-term memory network) model, which is another improved structure of the RNN model that shows superiority for solving the problems of gradient explosion.

## 4. Empirical Analysis and Discussion

### 4.1. The Data

This article selects the continuous futures contract of EUAf (European Union Allowance future, EUAf) from the European Energy Exchange as the representative variable of carbon assets. The data range from 22 June 2012 to 7 May 2021, with a total of 2274 data samples. The sample selection rule refers to the experience of Wen et al. [42], that is, continuous futures contracts with different maturity dates are connected according to the time sequence. Based on this, the sample of this article integrates the daily settlement price of the four futures contracts, DEC12, DEC16, DEC18 and DEC20. The reason for choosing EUAf is that the EUAf is the largest emission reduction quota in the world. The carbon futures trading of the European Energy Exchange accounts for about 70% of the global futures trading, and the EUAf trading volume is larger than EUAs (European Union Allowance spot, EUAs), the price discovery function is also relatively mature. It uses *R_t_* to represent the carbon assets return:

(17) $$R_{t}=100\times (lnP_{t}-lnP_{t-1})$$

where *P_t_* represents the carbon asset price, that is, the daily settlement price of EUAf continuous futures contracts.

### 4.2. Time-Varying High-Order Moment Characteristics Estimate

The study findings shown in Table 1 concluded that the ARCH and GARCH terms of all constant and time-varying high-order moment models are significant, indicating that the carbon return has obvious volatility clustering, which is not only caused by the variance shock, but also conditional skewness and conditional kurtosis, representing the impacts of asymmetric information and extreme factors on carbon return. All the volatility leverage coefficients $\beta_{3}$ are negative and significant, denoting that variance volatility has obvious asymmetry shock on carbon return, and the degree of negative impact is greater than the positive impact. This conclusion is completely consistent with the pioneering research results of Engle and Manganelli [43] that the negative VAR impact of the stock market is more significant. This finding indirectly proves that carbon assets have general financial attributes and common volatility characteristics.

The variance impact coefficient $\beta_{2}$ is smaller than the coefficient of the constant model, that is, with the addition of the conditional skewness and conditional kurtosis equations, the volatility clustering effect from the shock of variance term gradually decreases, for example, the $\beta_{2}$ coefficient of the GARCH, TGARCH and AGARCH models are 0.8824, 0.8875, 0.8904, respectively. When the time-varying conditional skewness and conditional kurtosis are added, the volatility clustering coefficients of AGARCH-K, NAGARCH-K and NAGARCHSK models are reduced to 0.8358, 0.8685, 0.7873, respectively. This conclusion is completely consistent with Harvey’s [39] research.

This phenomenon shows that when the time-varying, high-order moment models no longer assume the skewness and kurtosis are constants, they can effectively identify the volatility clustering effect caused by asymmetric information and extreme shocks through the time-varying skewness and kurtosis equation. We can say that the impact of carbon return from the time-varying skewness and kurtosis is becoming more obvious, resulting in the variance impact coefficient decreasing as the skewness and kurtosis coefficient increases. This similar reason can be used to explain why the extreme impact coefficient $\delta_{2}$ of the NAGARCHSK model is smaller than that of the AGARCH-K and the NAGARCH-K model.

**Figure 3:**
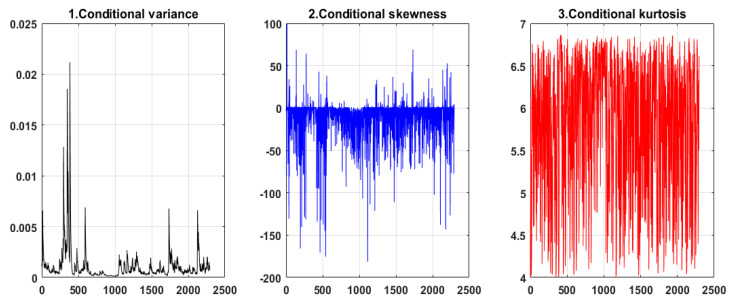
Time-varying, high-moment fluctuation of the carbon price identified by the NAGARCHSK model.

Compared with the constant model and other time-varying, high-order moment models, it should be noted that the maximum likelihood value of the NAGARCHSK model is the lowest, as shown in Table 1, therefore, this paper chooses the NAGARCHSK model to estimate the model parameters of time-varying conditional variance, conditional skewness and conditional kurtosis equations of the carbon return. Figure 3 shows that the conditional high-moment series of carbon assets have obvious volatility persistence effects, the risk of variance, skewness and kurtosis are large, and the high-moment volatility series also shows time-varying characteristics.

### 4.3. Predicting Results Analysis

We use the NAGARCHSK model to estimate the time-varying, high-order moment parameter characteristics that represent the shock from the asymmetric information and extreme external impact. Then, a multilayer GRU model is constructed to map and predict the carbon returns based on the obtained high-order moment series. The first 70% of samples of carbon return series are selected for model training, and the last 30% of samples for testing the prediction performance.

#### 4.3.1. GRU Structure Construction

Input unit, output unit, number of hidden layers and hidden layer neurons are the basic structure of a deep-learning network. The input of the carbon price forecasting NAGARCHSK-GRU model is the time-varying conditional lagging mean, conditional variance, conditional skewness and conditional kurtosis of carbon returns estimated by the NAGARCHSK model, and the output is the carbon return series we need to predict. The hidden layer is a network structure for parameter optimization and feature learning. Fewer hidden layers may limit the learning ability of the forecasting model, which makes it difficult to reach the optimal solution. Research has found that a neural network with two hidden layers can already solve most problems [44]. Similarly, the designing of hidden layer neurons is to capture and map the input data. Although more neurons can improve the learning and generalization ability of the network, it may also consume more training time and lead to overfitting.

For determining the appropriate the GRU network structure, based on the experimental method, this paper measures the forecasting performance when the hidden layers number is 1, 2, 3, 4, 5, 6 and the hidden layers neuron nodes are 4, 8, 16, 32, 64, 128, respectively (as showed in Table 2). It is found that when there are two hidden layers in the NAGARCHSK-GRU model, and the neuron nodes in both layers are 16-16, the model’s error criteria MSE, RMSE, and MAE values are 0.0006284, 0.0250681, and 0.1399925, respectively, which are the lowest of the whole experimental sample. Therefore, the network structure of the proposed NAGARCHSK-GRU forecasting model is designed as 4-16-16-1 for training the time-varying, high-order moment carbon series.

#### 4.3.2. Performance of the NAGARCHSK-GRU Model

For testing the prediction performance of the proposed NAGARCHSK-GRU model, this paper compares the prediction results of the proposed model and benchmark evaluation models. The results are shown in Table 3.

**Figure 4:**
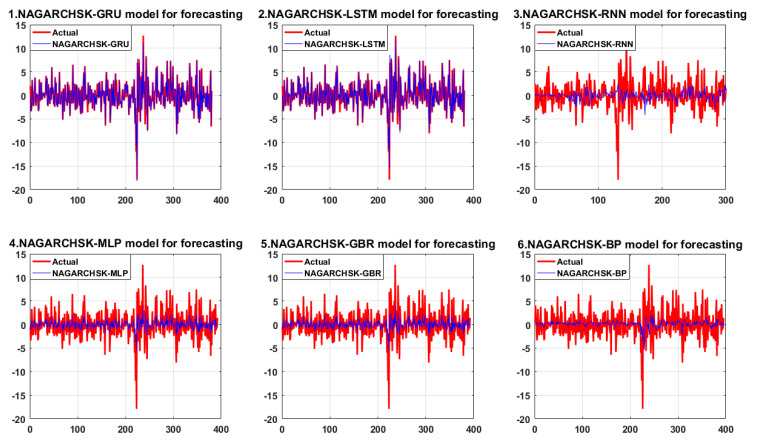
The forecasting performance of the proposed and benchmark model considering the features of time-varying conditional variance, conditional skewness and conditional kurtosis.

For high-order moment pricing models that consider time-varying conditional variance, conditional skewness and conditional kurtosis in Panel A, the NAGARCHSK-GRU model has significant advantages over other benchmark models in all the error evaluation criteria and market expected criteria. That is, the NAGARCHSK-GRU model has better prediction ability than other benchmark models (as shown in Figure 4).

Specifically, as for the error evaluation criteria, the RMSE, MAE, and MAPE values of the NAGARCHSK-GRU model are 0.509902, 0.172333, and 0.594527, respectively, which are lower than those of benchmark models such as NAGARCHSK-LSTM, NAGARCHSK-RNN, NAGARCHSK-MLP, NAGARCHSK-GBR, and NAGARCHSK-BP. This result concludes that the NAGARCHSK-GRU model has better robustness and stability for fitting carbon price series with time-varying, high-order moment characteristics. For the market-expected criteria, the DA of the NAGARCHSK-GRU model was 0.984211, which is higher than that of the benchmark models NAGARCHSK-LSTM (0.978947), NAGARCHSK-GRB (0.877551), NAGARCHSK-MLP (0.875), NAGARCHSK-RNN (0.742475) and NAGARCHSK-BP (0.729114). This indicates that the NAGARCHSK-GRU model is in line with investors’ psychological expectations for predicting carbon return, and the predicted returns are strongly consistent with the real return. As a result, the pricing model can provide technical support for investors to judge market conditions and formulate investment strategies.

In contrast, the error-evaluation criteria and market-expected criteria of the NAGARCHSK-RNN model shows the worst prediction effect of all models, that is, the RMSE, MAE and MAPE values are, respectively, 3.348075, 2.413839 and 7.415169, and the DA value is 0.742475. We can conclude that using the NAGARCHSK-RNN model it is difficult to map the carbon price series with the time-varying, high-order moment feature, and its predictive ability cannot meet investors’ expectations.

**Figure 5:**
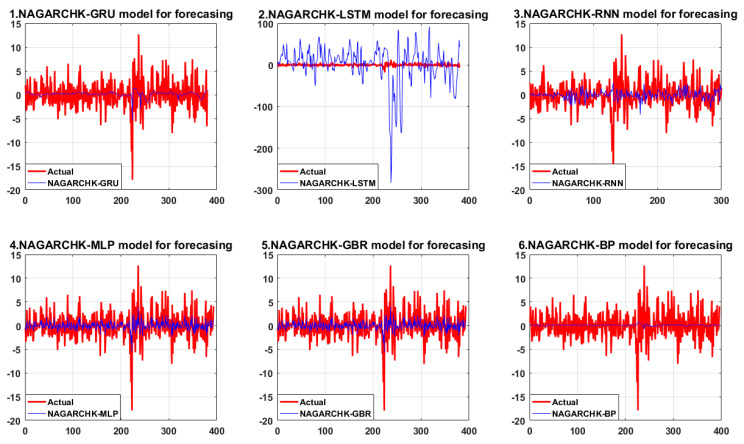
The forecasting performance of the proposed and benchmark models consider the feature of time-varying conditional variance and conditional kurtosis.

For the high-order moment forecasting models considering time-varying conditional variance and conditional kurtosis, as shown in Panel B, the NAGARCHSK-GRU model still has obvious forecasting advantages in error evaluation criteria and is relatively better in market-expected criteria compared with other benchmark models. Specifically, the error indexes RMSE, MAE and MAPE of the NAGARCHSK-GRU model are 2.034801, 1.473751 and 0.835218, respectively, which are lower than other benchmark criteria, the market-expected criteria DA is 0.731621, which is second only to the 0.843725 of the NAGARCHSK-MLP model. It is worth noting that the NAGARCHSK-LSTM model, which has the advantage in fitting financial time series, has the worst performance in carbon prediction among all models. The error criteria RMSE, MAE and MAPE are 4.616283, 2.380641 and 3.237167, respectively, and the market-expected criteria DA is 0.505615. This shows that the NAGARCHSK-LSTM model’s prediction ability and generalization ability are declining. The conclusion that the gap between the fitting curve and the real value is extremely obvious is also shown in Figure 5, and the correlation is poor.

For the carbon pricing model without considering the shock of time-varying, high-order moment, the GRU network model has the smallest error evaluation criteria (as shown in Panel C), which denotes that the GRU model still has strong prediction accuracy and robustness even without considering the characteristics of time-varying, high-order moment. However, the market-expected criteria DA is 0.74368, which is only higher than the 0.536842 of the LSTM model. We can conclude that the prediction performance of the model makes it difficult to satisfy the investors’ psychological expectations.

**Figure 6:**
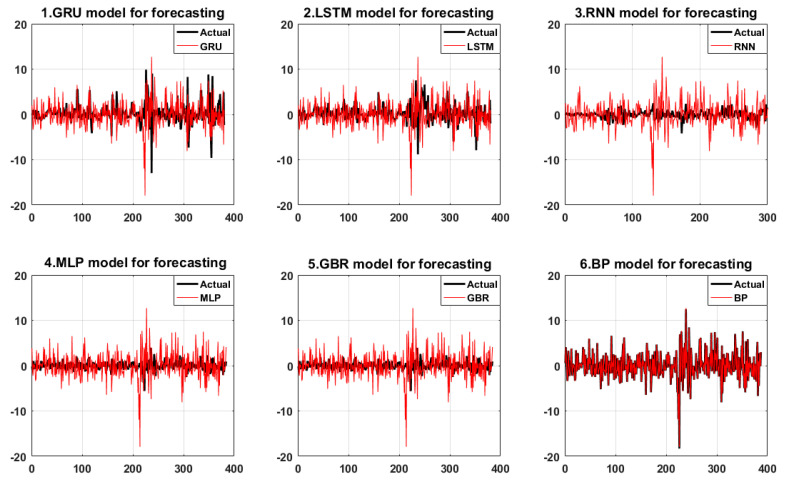
The forecasting performance of the proposed and benchmark models without considering the feature of time-varying, high-order moment.

Although the error criteria of other benchmark models are lower than those of the GRU model, the difference is not significant (as shown in Figure 6), particularly the RMSE and MAE of all models are basically close, and the deviation is small. More obviously, the market-expected criteria of the GBR and MLP models are significantly higher than those of other models, with DA values of 0.875318 and 0.872774, respectively, indicating the carbon-prediction performance of those two models is relatively stable and has a certain robustness. The market prediction performance suggests a reliable reference for investors making investment decisions.

Comparing the prediction results of all the pricing models of Panel A, Panel B and Panel C in Table 3, firstly, the carbon price-forecasting performance of the NAGARCHSK-GRU model is the best among all pricing models in Panel A, Panel B and Panel C. Secondly, the error criteria RMSE, MAE, and MAPE of the carbon pricing model in Panel A are significantly smaller than those of the error criteria in Panel B and Panel C, while the market-expected indicator DA is significantly higher than other models. Furthermore, the error criteria RMSE, MAE, and MAPE of the pricing model in Panel C are relatively high, while the market-expected index DA is relatively low.

The empirical results shown in Table 3 conclude that the deep-learning, carbon price forecasting model that considers the time-varying, high-order moment characteristics can provide more confident carbon premium evidence. This conclusion further proves that the carbon return is not only affected by the low-order moment attribute pricing factor, but also that the time-varying, high-order moment attribute that reflects the market asymmetric information and extreme shock is also an important explanatory factor for carbon return. The research results of this article can provide valuable reference for investors, commercial banks, and emission-reduction companies to judge market conditions and predict market trends.

#### 4.3.3. Robustness of the NAGARCHSK-GRU Model 

The significant advantage of the GRU model is that the parameter training structure of the long memory function can fit the finance time series, especially the time series over a long period time. Therefore, to prove the robustness of the NAGARCHSK-GRU model in different prediction period, this paper analyzes the performance of the proposed pricing model in the short-term, medium-term and long-term, respectively. Among them, the last 4 months, 10 months, and 15 months of the carbon returns are used as the prediction set in the short-term, medium-term, and long-term, respectively, and the rest of the data are used as the training set. The pricing model structure adopts the optimal network structure decided in the previous section. This part mainly describes the prediction performance of the carbon price-forecasting model that considers the time-varying, high-order moment characteristics, furthermore, the RMSE, MAE, MAPE error criteria are used to evaluate the model’s pricing accuracy and stability.

**Figure 7:**
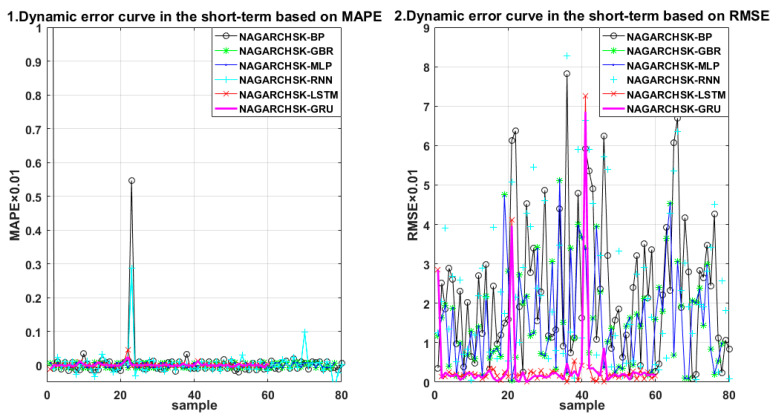
Error scatter distribution of the proposed and benchmark forecasting models considering the feature of time varying, high-order moment in the long term.

**Figure 8:**
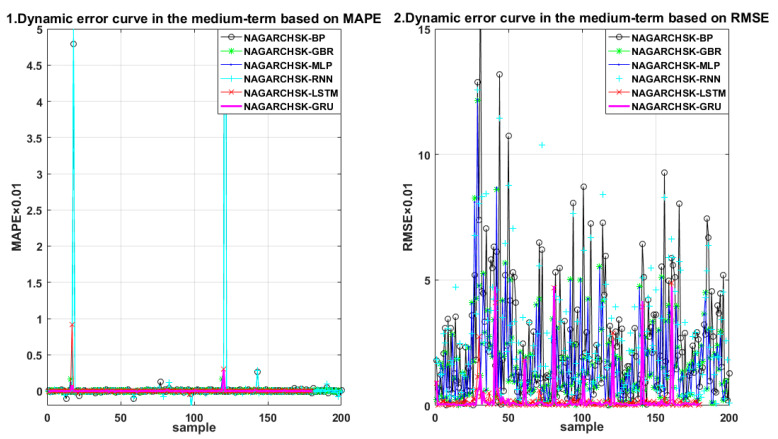
Error scatter distribution of the proposed and benchmark forecasting models considering the feature of time-varying, high-order moment in the medium term.

**Figure 9:**
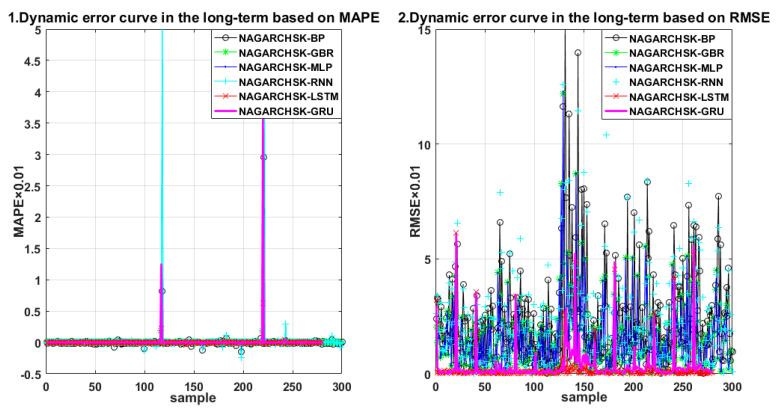
Error scatter distribution of the proposed and benchmark forecasting models considering the feature of time-varying, high-order moment in the short term.

For carbon price-forecasting performance in different periods (as shown in Table 4), the NAGARCHSK-GRU pricing model has significant superiority in the short-term, medium-term and long-term for all the error criteria, that is, the values of RMAE, MAE and MAPE are significantly lower than other benchmark models, and the proposed model has satisfactory robustness over all periods. The error distribution of the proposed and benchmark pricing models can be seen in Figure 7, Figure 8 and Figure 9.

Based on the estimation errors of the pricing models over different periods, it is found that as the forecasting period gradually increases from short-term to long-term, the forecasting errors of all pricing model gradually decrease, resulting the improvement of model accuracy and stability.

In particular, the NAGARCHSK-GRU model has the smallest prediction error and the best prediction performance, that is, the long-term prediction error RMSE, MAE and MAPE are 0.752385, 0.218883 and 0.354984, respectively, the medium-term prediction error RMSE, MAE and MAPE are 0.825573, 0.246388 and 0.476214, respectively, and the short-term prediction error RMSE, MAE and MAPE are 1.109585, 0.408066 and 0.264886, respectively. This evidence shows that the accuracy and stability of the NAGARCHSK-GRU model are gradually optimized with the extension of forecasting time, and it is significantly better than other benchmark models for forecasting the 15 month lagged returns. Since the advantage of the GRU model is fitting the longer finance time series, the findings of this article provide further evidence for this convinced conclusion and also the robust performance of the proposed model for different prediction periods.

## 5. Conclusions and Prospects

### 5.1. Conclusions

As a market-oriented mechanism for innovation to curb global climate issues, the carbon market is recognized as the most effective means to reducing the global carbon dioxide emissions and realize the sustainability of human society and economic growth. Compared with other financial markets, the carbon market has obvious market asymmetry, is sensitive to policy shocks and has time-varying volatility. However, the existing carbon price-forecasting research mainly focuses on the price information transmission and risk volatility spillover from the perspective of low-order moment of return, and ignores the time-varying impact of asymmetric information and extreme policies on carbon assets from the perspective of high-order moment attributes (market skewness and kurtosis). The explanation for carbon premium lacks sufficient evidential support. 

The innovation and contribution of this article are constructing an integrated carbon price-forecasting model, NAGARCHSK-GRU, based on the special characteristics of carbon assets such as market asymmetry, strong policy-shock sensitivity, and time-varying volatility. The proposed forecasting model considers the time-varying impact of market asymmetric information and extreme factors on carbon prices from the perspective of high-order moment attributes, so as to provide new evidence to explain the carbon premiums. The main work and research conclusions of this paper are as follows:

Firstly, carbon assets have obvious time-varying, high-order moment volatility characteristics. Compared with constant high-order moment volatility models, the time-varying, high-order moment volatility NAGARCHSK model can reveal the time-varying impact of systemic risk, asymmetric information and extreme factors on carbon premium by the function of time-varying variance, time-varying skewness and time-varying kurtosis equations. Moreover, the time-varying, high-order moment characteristics estimated by the NAGARCHSK model can explain the volatility clustering and premium mechanism of carbon price.

Secondly, the proposed machine-learning pricing model has more accuracy and stability in predicting carbon price with time-varying, high-order moment volatility characteristics. The time-varying impact of asymmetric information and extreme factors on carbon price is also important evidence for explaining carbon premium. This conclusion shows that the carbon pricing model proposed in this paper can fit and forecast carbon return effectively, specifically, the NAGARCHSK-GRU model is significantly better than other deep-network models. Further research shows that the NAGARCHSK-GRU model has reliable advantages in long-term, medium-term and short-term carbon price fitting and forecasting. In particular, the long-term carbon price-forecasting ability is outstanding, that is, it has perfect stability and accuracy for 15 months of prices forecasting. This conclusion not only confirms the advantages of the NAGARCHSK-GRU model in fitting long-term financial data, but also proves that the carbon pricing model considering time-varying, high-order moment volatility can provide a strong explanation for carbon price. 

The theoretical and practical implications of this paper are: firstly, as for the theoretical innovation, the findings show the rationality and effectiveness of incorporating the time-varying impact characteristics of asymmetric market information and extreme factors into the carbon price-forecasting model. The accuracy of carbon price forecasting can suggest a stronger reference for investors to judge market conditions, formulate investment strategies and may serve the implementation of carbon emission reduction. Secondly, as for the practical function, the maturity of carbon pricing mechanisms provide a decision-making basis for the government to speed up the construction of carbon market mechanisms and enhance the ability of the financial system to manage climate change. The conclusion of this paper also provides technical support for investors, emission-reduction entities and other market participants to capture price information and predict price changes.

### 5.2. Prospects

The focus of this paper is carbon premium explanation from the perspective of high-order moments. In the proposed high-order-moment pricing framework, each pricing term is a statistically structured factor, and the actual meaning behind the statistical indicators of high-order-moment attributes is not clear. Based on this, employing text-mining technology to obtain unstructured carbon pricing data that represent investor sentiment, policy impacts and other pricing factors, rather than the statistical moment attributes, is a valuable avenue for continuing relevant research in the future.

## Figures and Tables

**Table 1:** Parameter estimation of the high-order moment volatility model for carbon return.

Coefficient	Constant High-Order Moment Volatility Model			Time Varying High-Order Moment Volatility Model		
	GARCH	TGARCH	NGARCH	AGARCH-K	NAGARCH-K	NAGARCHSK
ρ	0.0794 (3.744)	0.0981 (4.562)	0.0916 (4.317)	0.0007 (0.011)	0.0007 (1.708)	0.0247 (0.005)
$\beta_{0}$	0.0012 (2.985)	0.0054 (4.4745)	0.0046 (3.951)	0.0715 (4.245)	0.0000 (2.177)	0.0483 (0.001)
$\beta_{1}$	0.1182 (7.794)	0.0899 (9.737)	0.1173 (17.87)	0.1463 (2.456)	0.1313 (6.428)	0.0587 (4.302)
$\beta_{2}$	0.8824 (70.919)	0.8875 (191.69)	0.8904 (185.7)	0.8358 (41.65)	0.8685 (50.659)	0.7873 (2.204)
$\beta_{3}$		−0.0632 (4.911)	−0.0536 (2.442)	−0.0029 (3.031)	−0.003 (3.848)	−0.0600 (2.964)
$\gamma_{0}$						0.7990 (0.807)
$\gamma_{1}$						0.0214 (2.831)
$\gamma_{2}$						0.0198 (5.325)
$\delta_{0}$				0.6978 (0.012)	0.4452 (0.281)	0.0821 (0.064)
$\delta_{1}$				0.3063 (3.086)	0.4265 (2.821)	0.6562 (1.987)
$\delta_{2}$				0.5363 (3.161)	0.5698 (3.101)	0.201 (3.256)
Likelihood	5159.771	5070.972	5067.591	6469	6473	**4682**

Note: The bold indicates the model with the minimum maximum likelihood value and the best parameter estimation performance; the data in brackets indicate the t-statistic of parameter estimation of each model.

**Table 2:** Performance of the proposed NAGARCHSK-GRU: hidden layers and hidden nodes.

Hidden Layer	Node	NAGARCHSK-GRU			Hidden Layer	Nodes	NAGARCHSK-GRU		
		MSE	RMSE	MAE			MSE	RMSE	MAE
1	4	0.0010592	0.0325455	0.2575152	4	4	0.0007587	0.0275444	0.1993575
1	8	0.0007156	0.0267501	0.1769118	4	8	0.0006820	0.0261152	0.1653054
1	16	0.0007580	0.0275324	0.1745386	4	16	0.0007306	0.0270302	0.1982328
1	32	0.0011345	0.0336824	0.3124025	4	32	0.0005733	0.0239427	0.1742260
1	64	0.0013580	0.0368505	0.4301757	4	64	0.0007967	0.0282252	0.2044582
1	128	0.0026156	0.0511425	0.6136194	4	128	0.0011475	0.0338750	0.2910599
	Avg	0.0012735	0.0347506	0.3275272		Avg	0.0007815	0.0277888	0.2054400
2	4	0.0007035	0.0265229	0.1684342	5	4	0.0009507	0.0308342	0.2467976
2	8	0.0007153	0.0267458	0.1627419	5	8	0.0006662	0.0258103	0.1402034
2	16	**0.0006284**	**0.0250681**	**0.1399925**	5	16	0.0006943	0.0263491	0.1738529
2	32	0.0006848	0.0261686	0.1953299	5	32	0.0008011	0.0283045	0.2853657
2	64	0.0009183	0.0303032	0.2546803	5	64	0.0012432	0.0352584	0.2500270
2	128	0.0011984	0.0346173	0.4536587	5	128	0.0011692	0.0341942	0.3119049
	Avg	0.0008081	0.0282376	0.2291396		Avg	0.0009208	0.0301251	0.2346919
3	4	0.0008391	0.0289676	0.2116799	6	4	0.0007273	0.0269685	0.1978246
3	8	0.0007236	0.0268994	0.1764938	6	8	0.0007203	0.0268387	0.1932607
3	16	0.0008080	0.0284251	0.1900228	6	16	0.0006415	0.0253283	0.1762878
3	32	0.0008078	0.0284224	0.1932762	6	32	0.0006575	0.0256409	0.2317956
3	64	0.0011074	0.0332772	0.2451673	6	64	0.0009107	0.0301776	0.2643418
3	128	0.0016887	0.0410936	0.2753646	6	128	0.0013865	0.0372361	0.3278892
	Avg	0.0009958	0.0311809	0.2153341		Avg	0.0008406	0.0286984	0.2319000

Note: Bold numbers are the minimum MSE, RMSE and MAE, respectively.

**Table 3:** Performance of the proposed and benchmark model for forecasting the carbon price.

Proposed Model		Benchmark Model				
Panel A: Pricing model considering the features of conditional variance, conditional skewness and conditional kurtosis						
	NAGARCHSK-GRU	NAGARCHSK-LSTM	NAGARCHSK-RNN	NAGARCHSK-MLP	NAGARCHSK-GRB	NAGARCHSK-BP
RMSE	**0.509902**	0.546867	3.348075	2.031849	2.033564	3.000422
MAE	**0.172333**	0.205202	2.413839	1.470269	1.471746	2.183393
MAPE	**0.594527**	1.219059	7.415169	0.705615	0.881136	3.450703
DA	**0.984211**	0.978947	0.742475	0.875	0.877551	0.729114
Panel B: Pricing model considering the features of conditional variance and conditional kurtosis						
	NAGARCHK-GRU	NAGARCHK-LSTM	NAGARCHK-RNN	NAGARCHK-MLP	NAGARCHK-GRB	NAGARCHK-BP
RMSE	2.034801	4.616283	3.347897	2.867784	2.036477	2.972087
MAE	1.473751	2.380641	2.413814	2.118404	1.475259	2.149908
MAPE	0.835218	3.237167	3.198746	3.293093	0.879472	2.526902
DA	0.731621	0.505615	0.583471	0.843725	0.715412	0.762763
Panel C: Pricing model without considering the feature of time-varying, high-order moment						
	GRU	LSTM	RNN	MLP	GBR	BP
RMSE	3.103279	3.623880	3.348127	3.107803	3.187022	3.822661
MAE	2.129058	2.434801	2.413955	2.261274	2.261036	2.194043
MAPE	7.316744	11.140187	7.395187	8.082685	8.035241	2.827125
DA	0.74368	0.536842	0.74	0.872774	0.875318	0.746231

Note: Bold numbers are the minimum MSE, RMSE, MAE, respectively, and the maximum of DA.

**Table 4:** Prediction performance of the pricing model considering the feature of time-varying, high-order moment.

Proposed Model		Benchmark Model				
	NAGARCHSK-GRU	NAGARCHSK-LSTM	NAGARCHSK-RNN	NAGARCHSK-MLP	NAGARCHSK-GRB	NAGARCHSK-BP
Panel A: Long-term prediction performance (15 months)						
RMSE	**0.752385**	1.157871	2.996408	1.981738	1.985327	3.001473
MAE	**0.218883**	0.412011	2.352625	1.559398	1.560740	2.393809
MAPE	**0.354984**	0.299397	0.765891	0.692497	0.689473	6.234842
Panel B: Medium-term prediction performance (10 months)						
RMSE	0.825573	0.877745	3.699027	2.388462	2.388496	3.686389
MAE	0.246388	0.345985	2.629891	1.698282	1.698174	2.590053
MAPE	0.476214	1.997452	10.473157	0.809171	0.838451	11.833577
Panel C: Short-term prediction performance (4 months)						
RMSE	1.109585	0.761311	3.348173	2.160763	2.162539	3.209178
MAE	0.408066	0.258531	2.414090	1.560627	1.562554	2.334026
MAPE	0.264886	0.873019	7.400078	0.794943	0.807474	2.576337

Note: Bold numbers are the minimum MSE, RMSE, MAE, respectively. The network structure of the proposed and benchmark models adopts the optimal structure decided experimentally in the previous section, that is, the structure of 4-16-16-1.

## Data Availability

The data in this paper are from the trading information of Environmental Markets in European Energy Exchange. The data can be obtained free of charge on the websites of Environmental Markets (eex.com). Of cause, the data are also available from the corresponding author upon reasonable request.
